# PDRN prevents SIRT1 degradation by attenuating autophagy during skin aging

**DOI:** 10.1371/journal.pone.0321005

**Published:** 2025-05-09

**Authors:** Jingjing Chen, Fanshan Qiu, Jianfeng Shi, Wei Huang, Chenyu Zhao, Qianqian Han

**Affiliations:** 1 State Key Laboratory of Cognitive Neuroscience & Learning and Ministry of Education Key Laboratory of Cell Proliferation & Regulation Biology, College of Life Sciences, Beijing Normal University, Beijing, China; 2 National Institutes for Food and Drug Control, Beijing, China; 3 Beijing Stomatological Hospital, Emergency Department, Beijing, China; Universita degli Studi della Campania Luigi Vanvitelli, ITALY

## Abstract

Polydeoxyribonucleotide (PDRN) is a low molecular weight linear polyribonucleotide fragment derived from salmon sperm, known for its potential in tissue regeneration and anti-inflammatory applications. However, its specific function in cellular senescence is yet to be fully understood. Silent information regulator 1 (SIRT1), an NAD + -dependent deacetylase, plays a crucial role in regulating cellular aging and tumorigenesis. Notably, SIRT1 levels decrease with age in both mice and during cellular senescence, highlighting its significance in anti-aging processes. This study assessed the effects of PDRN on cellular aging induced by ultraviolet B (UVB) or hydrogen peroxide (H_2_O_2_) and investigated the mechanisms of its protective effects against aging at the cellular level. Our data demonstrated that PDRN treatment mitigated the decline in cell viability and inhibited cell aging when exposed to UVB or H_2_O_2_. Furthermore, PDRN ameliorated UVB-induced epidermal thickening in mouse skin. Mechanically, we found that PDRN treatment led to a reduction in nuclear autophagy and the formation of cytoplasmic stress granules by preventing the accumulation of damaged LC3 in the nuclear and inhibiting the degradation of SIRT1 and p62 in the cytoplasm during cellular senescence. In conclusion, PDRN exhibits antioxidant and anti-aging properties by diminishing autophagy and enhancing SIRT1 expression. These results suggest that PDRN has potential as a therapeutic compound for reducing skin aging induced by UVB or H_2_O_2_ through the modulation of SIRT1 levels.

## Introduction

PDRN is an approved medication used in tissue repair and anti-inflammatory treatments [1,2]. The beneficial properties render it suitable for applications in regenerative medicine and wound healing, including treatments for diabetic foot ulcers and promoting angiogenesis [3,4]. Nevertheless, the anti-aging effects of PDRN have not been thoroughly investigated. The pharmacological actions of PDRN can be divided into two main categories: first, it contributes to the salvage pathway by supplying critical components for DNA synthesis in cells; second, it serves as an A2A receptor agonist, modulating the activation or inhibition of various signaling pathways and influencing downstream effects to produce diverse pharmacological outcomes [5]. Human skin aging, primarily caused by UV radiation, is a gradual and multifaceted process. Cellular senescence is similarly a complex biological phenomenon, influenced by various stressors such as UV exposure and oxidative stress from H_2_O_2_ [6,7]. These factors contribute to the accumulation of reactive oxygen species, DNA damage and dysregulated protein synthesis, subsequently activating key cellular senescence markers such as p16 (encoded by *INK4a*), p21 (encoded by *CDKN1A*) or p53 (encoded by *TP53*) [8,9]. Additionally, senescent cells exhibit an increased production of matrix metalloproteinases-01 (MMP1). Which leads to excessive degradation of collagen and elastin in the dermis, essential for maintaining skin integral. This degradation results in the appearance of wrinkles, loss of elasticity, and other aging signs [10]. Therefore, enhancing the skin’s antioxidant capacity using natural agents could effectively counteract oxidative damage from various sources. SIRT1, which deacetylates both histone and non-histone targets, is integral to various biological processes, including cellular senescence, and is involved in antioxidant, anti-apoptotic, and anti-inflammatory signaling pathways [11]. Research has indicated that SIRT1 undergoes shuttling between the nuclear and cytoplasm and is degraded via autophagosome-lysosome pathways in aged mice [12–14], suggesting that preventing SIRT1 degradation in the nuclear could represent a novel strategy to mitigate aging. However, the specific molecular mechanisms through which SIRT1 exerts its anti-aging effects on dermal fibroblasts exposed to UVB or H_2_O_2_ remain unclear. In this study, we investigated the potential reversal of cellular senescence induced by UVB or H_2_O_2_ through PDRN treatment. Our findings also highlighted a decrease in LC3 expression following PDRN treatment, suggesting that PDRN reduces the interaction between SIRT1 and LC3B, thereby preventing SIRT1 degradation through autophagy during the aging process. Overall, our research uncovers a new role for PDRN in UVB or H_2_O_2_ induced aging, likely by mitigating SIRT1 degradation linked to autophagy.

## Materials and methods

### Animal model

The mice (6–7 weeks old, weight 20–30 g) were purchased from the Institute of Laboratory Animal Resources, National Institutes for Food and Drug Control reviewed the study and approve the animal study protocol. Ethic approval number: FB2025009. The mice were divided into three groups: non-irradiated group (Control group, n = 3), irradiated group (UVB group, n = 3), irradiated +PDRN group (UVB+PDRN group, n = 3).UVB irradiation was measured as 200 mJ/cm^2^, once daily for five consecutive days for four weeks [6], PDRN was configured as a sterile solution at concentration of 800 mg/mL (intraperitoneally) and then injected into UVB-irradiated mice. After 21 days with PDRN treatment, the mice were administered ketamine (50 mg/kg) via intraperitoneal injection and the dorsal skin was harvested for subsequent assay. Animals were humanely euthanized via intravenous overdose of barbiturate to minimize distress.

### Preparation of paraffin sections

For paraffin sections, mouse skin specimens were fixed overnight in 10% formalin solution at 4 °C and were embedded in paraffin. Paraffin-embedded skin specimens were cut into 5-μm-thick. Paraffin sections were deparaffinized and then rehydrated and were stained with Haematoxylin and eosin(H&E) (Biosharp Life sciences)staining

### Cell culture, UVB or H_2_O_2_ dose and PDRN treatment

HaCaT (Human keratinocytes cells) were obtained from the Bioresource Collection and Research Center (BCRC, GCA, China) and were cultured in MEM-EBSS (MEM Eagles with Earle’s Balanced Salts) (Gibco). HDF (Human Dermal Fibroblast) cells were obtained from the Pricella (China) and were cultured in DMEF/Low Glucose (cytiva). Both were supplemented with 10% fetal bovine serum (FBS) (HyClone) maintained at 37°C and 5% CO_2_ in an incubator. Subsequently, the cells were exposed to 250 μM H_2_O_2_ for 24 h and incubated with PDRN dissolved in culture as reported [6]. For UVB treatment, cells were washed with PBS and exposed to UVB bulbs at an intensity of 300 mJ/cm^2^ [6,15]. After UVB exposure, the cells were treated with PDRN in dissolved in culture for 24 h recovery and then harvested.

### Antibodies and reagents

The antibodies used in the present study were: anti-GAPDH (TA-08, ZSGB-BIO), anti-p53 (clone PAb 122, Cat. No. 554147), anti-SIRT1 (8469S, Cell Signaling), anti-LC3 (L7543, Sigma-Aldrich), anti-p62 (Code No. PM045, MBL). The secondary anti-rabbit and anti-mouse antibodies were purchased from ZSGB-BIO, China. H_2_O_2_ was purchased from (St. Louis, MO, USA). PDRN was purchased from BlOOMAGE BIOTECH.

### CCK-8 assay

The cells were allowed to adhere for 24 h before exposure to UVB radiation and H_2_O_2_ treatment for five thousand cells were seeded into a 96-well plate every well. After UVB or H_2_O_2_ treatment, the cells were cultured in conditioned medium for 24 h. Cell viability was assessed by incubating the cells with 10μL CCK-8 reagent in every 96-well (#CA1210, Solarbio) for 2–4 h and the absorbance measured at 450 nm using a microplate reader (Molecular devices).

### Cell apoptosis

The identification of apoptosis was achieved through Annexin V and propidium iodide (PI) staining, followed by analysis via flow cytometry. Cells underwent a washing process using chilled PBS and binding buffer, followed by a re-suspension in the same buffer at a density of 1x106 cells per milliliter. The cell mixture was supplemented with Annexin V‐FITC and PI, followed by a 30-minute incubation in darkness. Right after labeling, the cells underwent analysis using flow cytometry. BD FACSVeres^TM^ Flow Cytometer was used to identify cells in early apoptosis (Annexin V‐positive, propidium iodide‐negative), necrotic/late apoptosis (double positive), and living cells (double negative).

### Wound assay

HaCaT cell suspension levels were modified to reach 1 × 10^6^ cells/mL and distributed into six-well plates. Following a 24-hour period, the supernatant was removed and the cells underwent a washing process. Cells underwent treatment with UVB or H_2_O_2_, succeeded by the introduction of a basic medium infused with PDRN. Additionally, an empty control group was established. Incubation of the culture plates occurred at 37 °C for 24-hour period. Images of cell scattering were captured using a microscope at intervals of 0, 12, and 24 hours following the introduction of PDRN. For every well, five fields of vision were chosen. Outcomes were derived from three high-power visual fields chosen at random, with average values computed across three replicated experiments. To calculate relative mobility, the formula used was: (initial scratch width-scratch width after culture)/initial scratch width × 100% [16].

### SA-β-Gal staining

The SA-β-gal staining kit (C0602, Beyotime) served to detect aging cells. HaCaT cells were cultured on slides over a 24-hour period and underwent various therapeutic methods. Cells underwent fixation using 4% paraformaldehyde at ambient temperature for a duration of 10 minutes before being cleansed with PBS. Following this, the cells underwent an overnight incubation at 37 °C in a staining solution. A light microscope was utilized to capture images.

### Immunoblotting

The cells underwent two washes with PBS and were disrupted using RIPA buffer (comprising 25 mM Tris-HCl, pH 7.6, 150 mM NaCl, 1% NP-40, 1% sodium deoxycholate, 0.1% SDS), enriched with 1 mM EDTA and a mixture of proteinase inhibitors (Roche 1183617001). Centrifugation of the lysates occurred at 15,000g for a duration of 15 minutes at a temperature of 4 °C. The clear liquid above the sediment was combined with a quadruple SDS loading solution and heated to 100 °C for a duration of 10–15 minutes. Following this, the samples underwent separation using 4–12% PAGE Bis-Tris gels before being moved onto PVDF membranes. Membranes underwent blocking using an additional buffer, followed by an overnight incubation at 4 °C with primary antibodies. The membranes underwent three washes using TBS-T (0.05% Tween) followed by additional incubation with secondary antibodies at a dilution of 1:1000. Visualization of the membranes was achieved through the use of a Tanon-5200 chemiluminescent system.

### Nuclear and cytoplasmic cell fractions

HaCaT cells were fractionated as previously described [17]. Briefly, HacaT cells were washed with cold PBS and lysised hypotonic buffer (10mM KCl, 10mM Tris-HCl pH 7.3, 1.5mM MgCl2, 1mM β-mercaptoethanol) containing PMSF and cocktails left on ice for 5 min. Then centrifuged for 15 min at 1,500 × g. The supernatant is harvested and mixed with 2% SDS for cytoplasmic fraction. The pellet is washed with a hypotonic solution supplemented with 100mM KCl and then centrifuged for 5 min at 1,500 × g. The supernatant is discarded and the pellet is resuspended in hypotonic buffer for nuclear fraction.

### Total RNA extraction and real-time polymerase chain reaction (RT-PCR)

The extraction of total RNA was conducted with Trizol reagent (Invitrogen, Carlsbad, CA, USA), adhering to the guidelines provided by the manufacturer. Utilizing the RevertAid First Strand cDNA Synthesis Kit (Thermo Scientific, K1621), the total RNA underwent reverse transcription into cDNA. The Quant Studio 6 Fast Real-time PCR system (Applied Biosystems) was utilized to conduct real-time RT-PCR. Table 1 shows the primer sequences utilized in real-time qPCR. Gene expression levels were standardized in relation to GAPDH levels.

**Table 1:** Primer sequences used for RT-PCR.

Gene	Forward Primers (5’-3’)	Reverse Primers (5’-3’)
SIRT1	TGACTTCAGGTCAAGGGATGG	GGGAAGTCTACAGCAAGGCG
p53	TCTCCCTCACTGGAACAAGC	ACCTGCACCTCCTGACTAAA
p21	TGAGCCGCGACTGTGATG	GTCTCGGTGACAAAGTCGAAGTT
p16	CACCGAATAGTTACGGTCGG	GCACGGGTCGGGTGAGAGTG
GAPDH	GGAGCGAGATCCCTCCAAAAT	GGCTGTTGTCATACTTCTCATGG

### Immunofluorescence

The cells underwent cultivation within six-well chamber slides. Following this, the cells underwent fixation using 4% paraformaldehyde in PBS for 10 minutes, were made permeable with 0.2% Triton X-100 in PBS for 5 minutes, and then obstructed with 3% BSA for an hour. Subsequently, the cell specimens were incubated overnight at 4 °C with primary antibodies aimed at specific antigens in the blocking buffer. The specimens underwent three PBS washes and were then subjected to a one-hour room temperature incubation with host-specific Alexa Fluor 488/555/647 secondary antibodies (ZSGB-BIO). In the process of microscopic imaging, slides were prepared using a mounting solution that included DAPI (Thermo Fisher Scientific P36931). The imagery was taken through an Olympus confocal microscope equipped with a 60 × Oil objective.

### Statistical analysis

Unpaired two-tailed Student’s t-tests were conducted for comparisons between two groups. One-way ANOVA followed by Dunnett’s test multiple comparison test. All bar graphs represent the mean values with error bars (S.E.M., as indicated in the Fig legends). P value < 0.05 indicated statistical significance. The number of experimental replicates is indicated in the Figure legends.

## Results

### PDRN alleviates reduction of UVB or H_2_O_2_-induced proliferation and apoptosis

**Fig 1:**
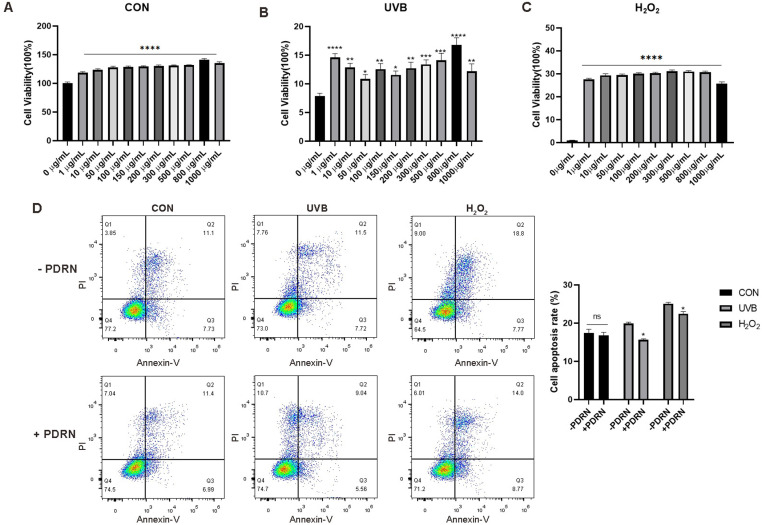
PDRN promotes proliferation and inhibits apoptosis of HaCaT cells. (A) PDRN promotes proliferation of HaCaT cells as demonstrated by CCK8 assay. (B-C) Percentage of apoptotic cells after HaCaT cells were exposed to UVB or H2O2 and then co-treated with various concentrations of PDRN and evaluated by CCK8 assay and flow cytometry. (D)Percentage of apoptotic cells after HaCaT cells were treated with 800 µg of PDRN for 24 h and stained with annexin V-FITC/propidium iodide (PI) and then evaluated by apoptosis assay and flow cytometry(d). Representative scatter plots of PI (y-axis) vs. annexin V (x-axis) staining. Percentage of apoptotic cells after exposed to H2O2 or UVB. Values represent means + S.E.M of three independent experiments (n = 3). * p < 0.05, ** p < 0.01, and *** p < 0.001,**** p < 0.0001vs. untreated control cells.

HaCaT cells were subjected to varying concentrations of PDRN (0, 12.5, 25, 50, and 100 μM) for 24 hours to assess the impact of PDRN on cell viability, which was measured using the CCK-8 assay. Although PDRN did not exhibit a dose-dependent increase in cell proliferation relative to the control (*p < 0.05), the optimal concentration was determined to be 800 μg/mL (Fig 1A). Further, we examined the effect of UVB or H_2_O_2_ on HaCaT cell proliferation. The cells were treated with H_2_O_2_ or exposed to UVB to induce aging, followed by treatment with various PDRN concentrations for 24 hours. The results indicated that higher concentrations of PDRN enhanced cell viability compared to the controls subjected to UVB or H_2_O_2_ (Fig 1B, C). These results imply that PDRN provides protection to HaCaT cells against UVB and cellular death. The subsequent experiments focused on the 800 μg/mL PDRN dose. The impact of PDRN on apoptosis was analyzed through flow cytometry after staining the cells with annexin V and PI. Annexin V identifies both early and late apoptotic cells, while PI staining is used for detecting late apoptosis or necrosis. Early apoptotic cells were characterized by positivity for annexin V and negativity for PI (lower right quadrant), while late apoptotic cells were positive for both (upper right quadrant) (Fig 1D). In comparison to the control group (4.0%), the proportion of late apoptotic cells rose to 7.0%, 13.0%, and 28.9% following UVB or H_2_O_2_ exposure after treatment with 800 μg PDRN within 24 hours. These findings indicate that PDRN significantly diminishes apoptosis triggered by UVB or H_2_O_2_.

### PDRN promotes migration and decreases positive SA-β-gal-staining

**Fig 2:**
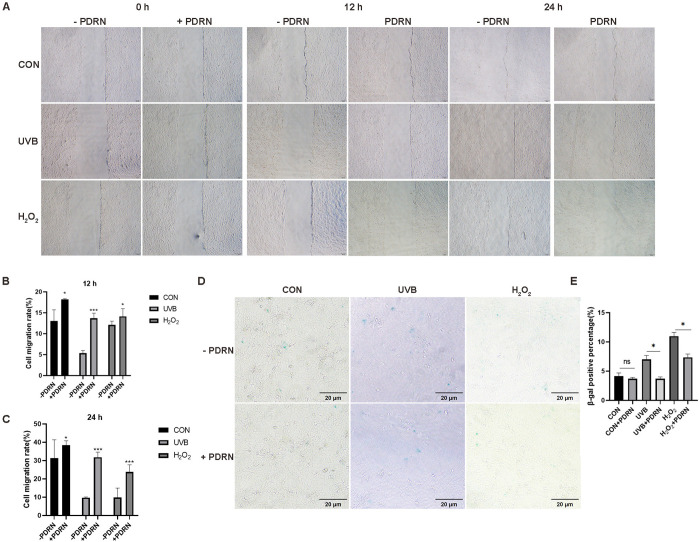
PDRN reverses senescence in HaCaT cells. (A) Migration of HaCaT cells at 12 h and 24 h after PDRN treatment was observed under a phase‑contrast microscope. Original magnification, x10. (B-C) The number of migrated PDRN‑treated HaCaT at 12 h and 24 h were counted. Values shown are means + S.E.M. Quantification of the results is shown (n = 3), * *p* < 0.05, ** *p* < 0.01, *** *p* < 0.001 vs. untreated control cells. **(D-E)** β-gal-stained cells at 24h post PDRN treatment were imaged by microscopy; n = 2 independent experiments.

Cell migration is crucial for repairing and regenerating skin tissue. UVB irradiation or H_2_O_2_ treatment leads to skin aging [18,19]. Previous studies have shown that PDRN accelerates the healing of damaged skin wounds [2]. Therefore, we investigated whether PDRN could enhance the migration of HaCaT cells subjected to UVB or H_2_O_2_ stress. Following PDRN treatment, the migration rate of the treated cells was significantly greater than that of the control group at 12–24 hours (Fig 2A–C). Likewise, the migration of cells exposed to UVB or H_2_O_2_ was augmented by PDRN treatment. To evaluate cellular aging, we performed senescence-associated β-galactosidase (SA-β-gal) staining. Quantitative analysis demonstrated that UVB or H_2_O_2_ treatment significantly increased the proportion of β-gal-positive senescent cells compared to control groups (Fig 2D, E). Conversely, PDRN treatment mitigated keratinocyte senescence induced by UVB or H_2_O_2_. These findings suggest that PDRN boosts skin cell migration, likely by enhancing the expression of growth factors, thereby improving wound healing. These results imply the vital role of PDRN in tissue repair, combating photoaging, and restoring skin health, ultimately contributing to anti-aging effects.

### PDRN attenuates SIRT1 stress granules in the cytoplasm

**Fig 3:**
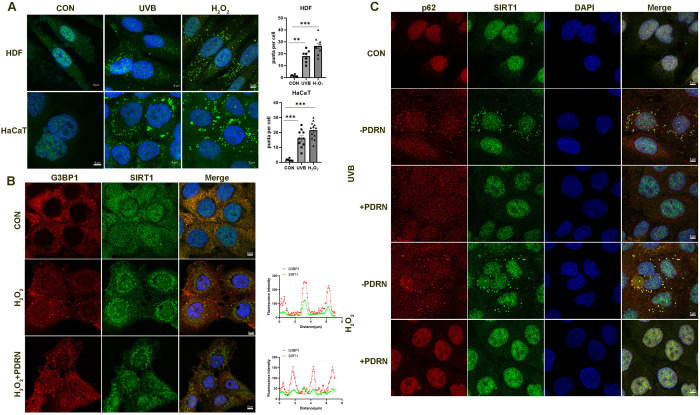
PDRN upregulates SIRT1 protein expression in HaCaT cells exposed to UVB or H_2_O_2_. (A) Immunostaining of SIRT1 in the cytoplasm following UVB or H_2_O_2_ in HDF and HaCaT cells. The green (endogenous SIRT1) and blue (DAPI) signals were merged to indicate cytoplasm co-localization. Graphs at the right panel represent the numbers of stress granules. scale bars, 5 µm. Values represent means + S.E.M of three independent experiments (n = 3). * *p* < 0.05, ** *p* < 0.01, vs. untreated control cells. (B) Immunostaining of G3BP1 co-localization with SIRT1 in the cytoplasm following H_2_O_2_. The green (endogenous SIRT1), red (endogenous G3BP1), and blue (DAPI) signals were merged to indicate cytoplasm co-localization. Graphs at the bottom of the right panel represent the relative signal intensity of the indicated white line: scale bars, 5 µm. Each graph represents the normalized fluorescence distribution over the white dashed lines. Representative results from two independent experiments. (C) Immunostaining of p62 co-localization with SIRT1 in the cytoplasm following exposure to UVB or H_2_O_2_. The green (endogenous SIRT1), red (endogenous p62), and blue (DAPI) signals were merged to indicate cytoplasm co-localization. scale bars, 5 µm. The white arrow indicates the location of the foci.

SIRT1 regulates cellular senescence and autophagy in various cell types under stress conditions [12,20], so we also researched the expression SIRT1 after treatment with PDRN. We found that under UVB or H_2_O_2_ treatment, SIRT1 forms stress granules in the cytoplasm by immunofluorescence in HaCaT and HDF cells (Fig 3A). Next, we found SIRT1 stress granules co-localization with G3BP1 when expose to H_2_O_2_, G3BP is stress granule marker [21]. However, PDNR could alleviate the stress granules when exposed to H_2_O_2_ (Fig 3B). Meanwhile, we also investigated that SIRT1 could also co-localization with autophagy substrate p62.It is reported that p62-gels are not simple substrates for autophagy but serve as platforms for both autophagosome formation and anti-oxidative stress [22].So we suppose SIRT1 forms stress granules in cytoplasm exposed to UVB or H_2_O_2_ for degradation, but PDRN could reverse SIRT1 degradation by decreasing the stress granules.

### PDRN attenuates the nuclear autophagy and upregulates SIRT1 expression

**Fig 4:**
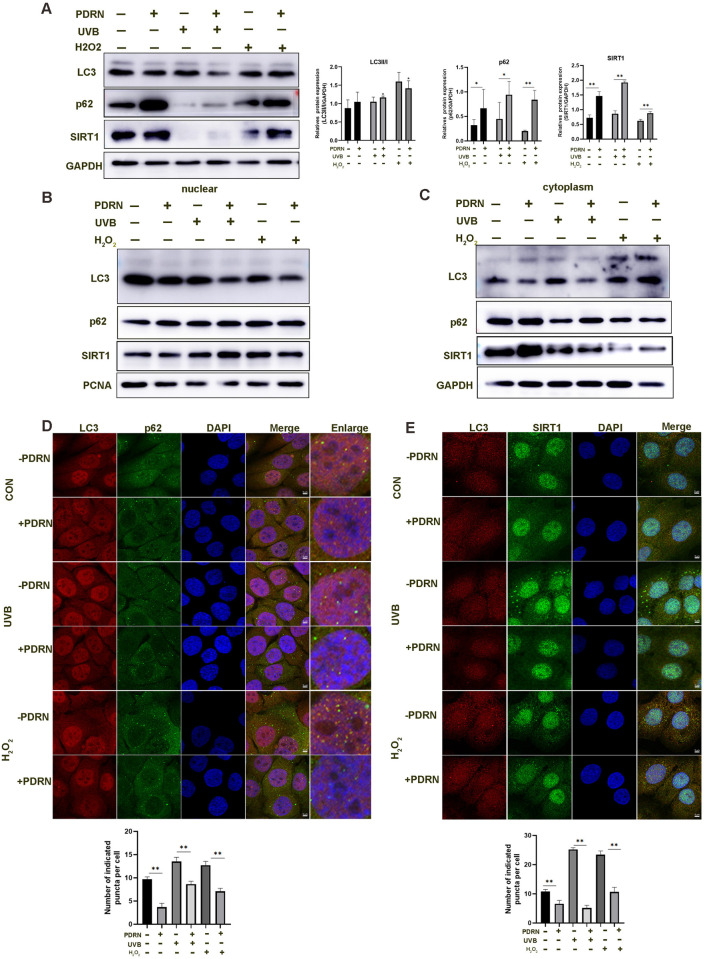
PDRN upregulates SIRT1 protein expression in HaCaT cells exposed to UVB or H_2_O_2_. (A) Levels of SIRT1, p62, and LC3 proteins in HaCaT cells exposed to UVB or H_2_O_2_ after treatment with PDRN at the optimal concentration as determined by Western blot. Values represent means + S.E.M of three independent experiments (n = 3). * *p* < 0.05, ** *p* < 0.01, vs. untreated control cells. (B-C) HaCaT cells were fractionated by hypotonic lysis and cytoplasmic and nuclear fractions were used for immunoblotting. PCNA and GAPDH proteins were detected as markers of nuclear and cytoplasmic. (D) Immunostaining of LC3 co-localization with p62 in the nuclear following exposure to UVB or H_2_O_2_. The green (endogenous p62), red (endogenous LC3), and blue (DAPI) signals were merged to indicate nuclear co-localization. Graphs at the bottom of the right panel represent the relative puncta amounts of the indicated white arrow: scale bars, 5 µm. Quantification is shown in right panel, unpaired two-tailed Student’s t-tests were conducted for comparisons between two groups. ** *p* < 0.01, vs. treated control cells. (E) Immunostaining of LC3 co-localization with SIRT1 in the cytoplasm following exposure to UVB or H_2_O_2_. The green (endogenous SIRT1), red (endogenous LC3), and blue (DAPI) signals were merged to indicate cytoplasm co-localization. Graphs at the bottom of the right panel represent the puncta of the indicated white arrow: scale bars, 5 µm. Quantification is shown in right panel, unpaired two-tailed Student’s t-tests were conducted for comparisons between two groups. ** *p* < 0.01, vs. treated control cells.

Studies have reported that SIRT1 interacted LC3 in the nuclear, but following cellular senescence, leading to the translocation of SIRT1 from nuclear to the cytoplasm and is degraded by autophagosome-lysosome [13]. Next, we observed by WB that PDRN increased the expression of SIRT1 and p62 protein, especially in UVB-induced photoaging or H_2_O_2_-oxidative stress. In addition, LC3II/I ratio decreased when cells were exposed to UVB or H_2_O_2_, but this effect was reversed following treatment with PDRN (Fig 4A). Next, we isolated cytoplasmic and nuclear fractions of HaCaT cells.PDRN promoted the protein expression of SIRT1 in the cytoplasm without UVB or H_2_O_2_, but the expression of SIRT1 in the nuclear and cytoplasm decreased with UVB or H_2_O_2_ treatment (Fig 4B, C). PDRN inhibited SIRT1autophagic degradation by decreasing the expression of LC3 in the nuclear and cytoplasm and increasing the expression of SIRT1, but there was no significant change of p62 in the nuclear, which implied that SIRT1 was the main substrate for the degradation of the nuclear consistent with the reported [13]. Furthermore, immunofluorescence results showed that PDRN attenuated the nuclear localization of LC3 and p62 by autophagy puncta. Notably, under UVB or H_2_O_2_ stress, LC3 and p62 accumulation was obvious observed in the nuclear, but administration of PDRN attenuated these puncta localization (Fig 4D), indicating that autophagy was impaired in the nuclear. We also investigated that PDRN attenuated localization LC3 and SIRT1 puncta accumulation in the cytoplasm when exposed to UBV or H_2_O_2_ (Fig 4E). In summary, PDRN impaired nuclear autophagy and prevented the degradation of SIRT1 in cytoplasm. Therefore, we speculate that PDRN decrease autophagy to attenuate SIRT1 degradation in the cytoplasm and increase the protein expression level of SIRT1 to counteract the onset of senescence.

### Inhibition of UVB or H_2_O_2_-induced cell senescence with treatment of PDRN

**Fig 5:**
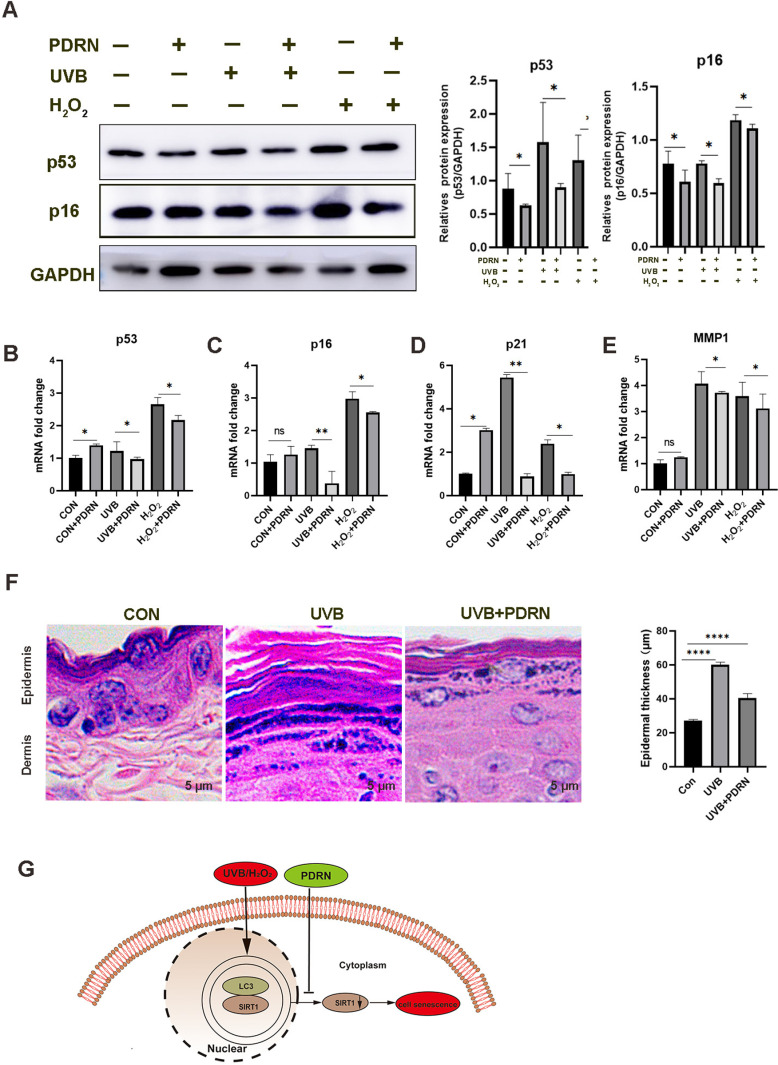
PDRN decreases the expression of cellular aging markers. (A) The protein expression of p53 and p16 were analysed by Western blotting. unpaired two-tailed Student’s t-tests were conducted for comparisons between two groups. *p < 0.05, vs. treated control cells. **(B-E)** mRNA levels of SIRT1, p53, and p21, p16 and MMP-1 in HaCaT cells exposed to UVB or H_2_O_2_ and treated with PDRN (800 μg/mL) as determined by real-time PCR. Values represent means + S.E.M of three independent experiments (n = 3), * *p* < 0.05, ** *p* < 0.01 vs. untreated control cells. (F) Representative H&E images of the skin scale of CON, UVB and UVB+PDRN mice. Quantification of epidermal thickness in skin from the difference mice, values represent means + **S.**E.M and were analysed with Dunn’s multiple comparison. (right panel). (G) Scheme displaying the inhibition of UVB or H_2_O_2_-induced skin aging damage by PDRN.

To further identify whether exposure to UVB or H_2_O_2_ causes dermal aging. We investigated the expression of aging markers, such as p53, p21 and p16 [23]. Immunoblotting showed that p53 and p16 protein level decreased after treatment of the cells with PDRN (Fig 5A), implying PDRN damaged the overactivity of p53 and protect cells against oxidative damage. Furthermore, mRNA analysis was performed to investigate the anti-aging effect of PDRN. Our results demonstrated that aging markers, such as p53, p21, and p16, were upregulated following exposure to UVB or H_2_O_2_ at mRNA level (Fig 5B–D). However, these results were reversed by PDRN treatment, implying PDRN make antiaging when exposed UVB or H_2_O_2_. Matrix metalloproteinase-1 (MMP1), which initiates the cleavage of collagen fibrils and hydrolyze collagen protein, is significantly increased in aged human skin [24]. PDNR reduces MMP-1 expression in human skin fibroblast cells [25].We also investigated that PDRN reduces MMP-1 mRNA expression in HaCaT cells when exposed to UVB or H_2_O_2_ (Fig 5E), To further demonstrate the role of PDRN in vivo, irradiated the skin of mice with UVB, and HE staining revealed that UVB irradiation induced skin atrophy, a decrease in the number of epidermal cell layers, and a flattening of the basal cellular morphology in the mice, which were similar to the aging characteristics of human skin [6], but PDRN improved these aging characteristics of the skin (Fig 5F).So it is further uncovered that function of PDRN for skin aging when exposed to UVB or H_2_O_2_, confirming the effect of PDRN treatment for skin aging in vivo and vitro. In addition, we also make a scheme for our study (Fig 5G), in the basal state, PDRN attenuates the nuclear autophagy which damaged LC3 and SIRT1 interaction in the nuclear, upregulated the level of SIRT1. Upon UVB or H_2_O_2_-induced cell, LC3-SIRT1 interaction is enhanced, followed by nuclear-to-cytoplasm translocation of the LC3-SIRT1 complex, leading to cytoplasmic degradation of SIRT1 by autophagy, but PDRN can reverse these results and upregulate expression of SIRT1 for defending cellular senescence.

## Discussion

Skin aging is a complex process influenced by many factors, such as ultraviolet (UV)radiation from the sun and oxidative damage. In this study, we utilized UVB-induced photoaging or H_2_O_2_-induced oxidative stress models to explore the effects of PDRN on anti-aging. The findings revealed that keratinocytes exhibited significantly reduced cell proliferation and increased cell apoptosis when exposed to UVB or H_2_O_2._ Additionally, previous findings indicated that PDRN protects cells from UVB-induced DNA damage, potentially by enhancing p53 protein expression through the salvage pathway [26]. Therefore, we hypothesized that PDRN promotes cell proliferation by modulating the expression of key genes.

SIRT1 plays an essential role in antioxidant, anti-apoptotic and senescence processes [20,27,28]. In this study, PDRN significantly upregulated SIRT1 expression at protein levels and attenuates stress granules in cytoplasm, particularly under UVB or H_2_O_2_ stimulation, implying PDRN could decrease skin aging by promoting the expression of SIRT1. Immunofluorescence and nuclear or cytoplasmic cell fractions results showed that PDRN-treated cells exhibited significantly enhanced expression of SIRT1 and decreased localization of LC3 in the nuclear, indicating that PDRN reduced the interaction of SIRT1 and LC3B within the nuclear, thereby preventing SIRT1 degradation in the cytoplasm. Furthermore, p62, a substrate for autophagic degradation [29], was upregulated upon treatment with PDRN, especially in cytoplasm after treatment PDRN, further indicating a reduction in autophagy in the nuclear. This upregulation of SIRT1 expression may mitigate oxidative stress-induced senescence. Certain genes, such as p53, p21, p16, are overexpressed during senescence and can serve as senescence markers. Although previous findings indicate that PDRN can mitigate UVB-induced DNA damage by providing nucleotides and nucleosides as part of the salvage pathway, the precise mechanism through which it inhibits senescence requires further investigation [5]. Our findings further revealed that even without external stimulation, PDRN increased the transcriptional levels of p53 and p21, thereby promoting cell proliferation. However, upon UVB or H_2_O_2_ stimulation, the expression of these transcription factors were significantly upregulated. PDRN inhibited overexpression of these transcription factors, indicating its potential anti-aging properties. In addition, we observed PDRN downregulated the expression of MMP1 after UVB or H_2_O_2_ stimulation, implying that PDRN can modulate the collagen content and function as an anti-aging agent. Human skin aging is characterized by thinning, fragility, and hyperpigmentation [30]. In order to verify the anti-aging effect of PDRN on the skin in vivo, we administered UVB irradiation to mice to simulate the characteristics of human skin aging. H&E results showed that UVB irradiation induced skin atrophy, thinning of the epidermal layer, and cellular reduction in the skin of mice, but the aging skin could be restored to the same level as that of the control group after treatment with PDRN, implying that PDRN can greatly alleviate the skin photodegradation induced by UVB. In summary, this study provides the first evidence that PDRN can inhibit UVB or H_2_O_2_-induced senescence. Moreover, we observed that PDRN significantly upregulates the expression of SIRT1 and attenuates nuclear autophagy, thus offering a preliminary understanding of its anti-aging mechanism.

## Conclusions

This study demonstrates that PDRN effectively inhibits the aging of keratinocytes cells by upregulating SIRT1 expression and reducing nuclear autophagy when exposed to UVB or H_2_O_2_-induced senescence including in mice skin. Additionally, we observed that PDRN attenuates UVB or H_2_O_2_-induced senescence by downregulating the expression of specific senescence-associated markers such as p53, p21 and p16. These findings reveal the cellular anti-aging properties of PDRN and provide insights into the mechanisms underlying its potential as a natural ingredient for developing skincare products.

## Supporting information

S1 Data Raw data images of the original Western blotting images on the PVDF membrane, staining with immobilon with HRP substrate detected by Tanon 5200. (PDF)
