# Supplementary figures and images for: PDRN prevents SIRT1 degradation by attenuating autophagy during skin aging
Source: PLoS One. 2025 May 9;20(5):e0321005. doi: 10.1371/journal.pone.0321005 (PMC12063799; doi:10.1371/journal.pone.0321005)

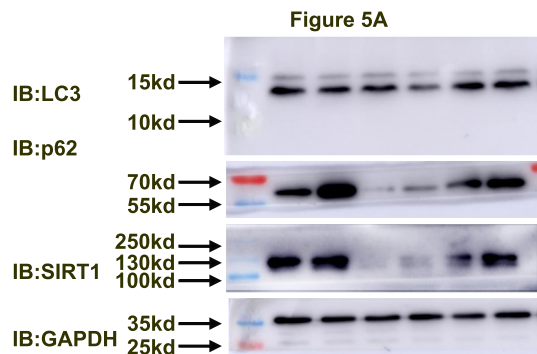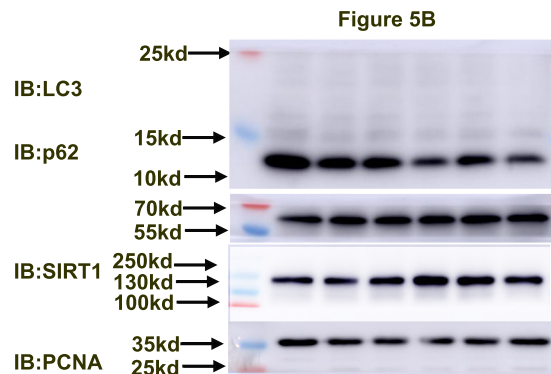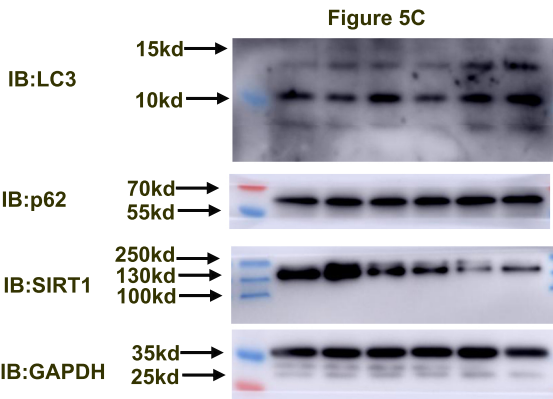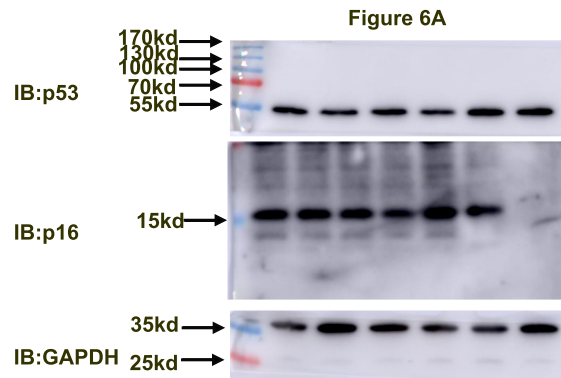

Supplement: S1 Data — (PDF) [file pone.0321005.s001.pdf]
